# Supplementary material for: Reconciling Mining with the Conservation of Cave Biodiversity: A Quantitative Baseline to Help Establish Conservation Priorities
Source: PLoS One. 2016 Dec 20;11(12):e0168348. doi: 10.1371/journal.pone.0168348 (PMC5173368; doi:10.1371/journal.pone.0168348)
Supplement: S1 Dataset — (ZIP) [file pone.0168348.s002.zip › Taxa/Serra Norte/SN_2013/N5S/Lista N5S-81.pdf]

CAVIDADE N5S-0081

Classe	Ordem	Fam/Outros	Gên/Outros	Espécie	S-U	U-S	Zon.
Annelida	Oligochaeta			jovem	2		E
Arachnida	Amblypygi	Phrynidae	*Heterophrynus*	*longicornis*	2	4	E
Arachnida	Araneae	Corinnidae		jovem	1		E
Arachnida	Araneae	Ctenidae		jovem	2		E
Arachnida	Araneae	Pholcidae	*Mesabolivar*	*aurantiacus*	X	X	E
Arachnida	Araneae	Pholcidae	*Mesabolivar*	sp.1	X		E
Arachnida	Araneae	Pisauridae	*Architis*	sp.1		1	E
Arachnida	Araneae	Scytodidae	*Scytodes*	sp.1		1	E
Arachnida	Araneae	Theridiosomatidae	*Plato*	sp.1	X	X	E
Arachnida	Opiliones	Sclerosomatidae	*Prionostema*	sp.1	2		E
Diplopoda	Polydesmida	Paradoxosomatidae		sp.1		1	E
Entognatha	Collembola	Entomobryidae		sp.1		X	E
Entognatha	Collembola	Entomobryidae		sp.5	X		E
Insecta	Blattodea			jovem	1	1	E
Insecta	Coleoptera	Carabidae		sp.2		X	E
Insecta	Coleoptera	Carabidae		sp.10	X		E
Insecta	Coleoptera	Dytiscidae		sp.3		X	E
Insecta	Coleoptera	Staphylinidae		sp.4	X		E
Insecta	Coleoptera	Staphylinidae		sp.24	X		E
Insecta	Diptera	Culicidae	*Culex*	sp.		X	E
Insecta	Diptera	Psychodidae	*Breviscapus*	sp.		X	E
Insecta	Diptera	Psychodidae	Phlebotominae	sp.		X	E
Insecta	Diptera	Tipulidae	Tipulinae	sp.	X	X	E
Insecta	Heteroptera	Veliidae	*Paravelia*	sp.	X	X	E
Insecta	Homoptera	Cixiidae		jovem		X	E
Insecta	Hymenoptera	Formicidae	*Paratrechina*	sp.1		X	E
Insecta	Lepidoptera	Arctiidae		sp.	X		E
Insecta	Lepidoptera	Noctuoidea		sp.3	1		E
Insecta	Orthoptera	Gryllidae		sp.		1	E
Insecta	Orthoptera	Phalangopsidae	*Paraclodes*	sp.	1		E
Insecta	Orthoptera	Phalangopsidae	*Phalangopsis*	sp.	3	10	E
Insecta	Psocoptera	Epipsocidae	*Epipsocus*	sp.1	X		E
